# Supplementary material for: Defective Lysosomal Lipolysis Causes Prenatal Lipid Accumulation and Exacerbates Immediately after Birth
Source: Int J Mol Sci. 2021 Sep 27;22(19):10416. doi: 10.3390/ijms221910416 (PMC8508985; doi:10.3390/ijms221910416)
Supplement: Supplementary file 1 [file ijms-22-10416-s001.zip › ijms-1377505-supplementary.pdf]

SUPPLEMENT

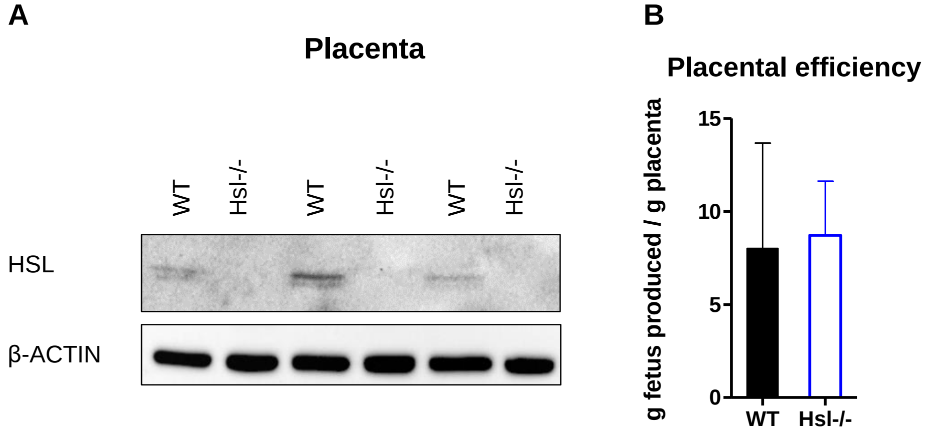

Figure S1. HSL deficiency does not alter placental efficiency. (**A**) Western blot analysis of placental tissue. (**B**) Placental efficiency of WT and Hsl-/- mice (n = 34 WT / 24 Hsl-/-). Data represent mean values + SD.

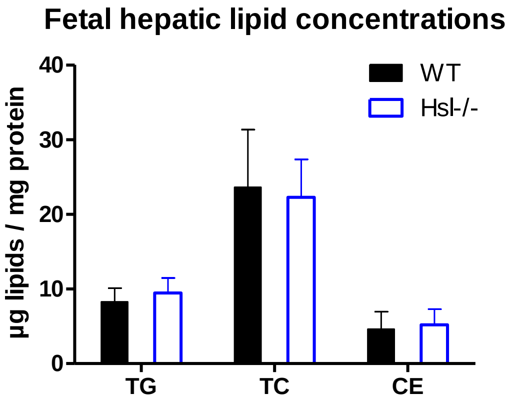

Figure S2. HSL deficiency does not alter hepatic lipid concentrations in the fetus. Fetal hepatic triglyceride (TG), total cholesterol (TC), and cholesteryl ester (CE) levels. Data represent mean values + SD (n= 4).

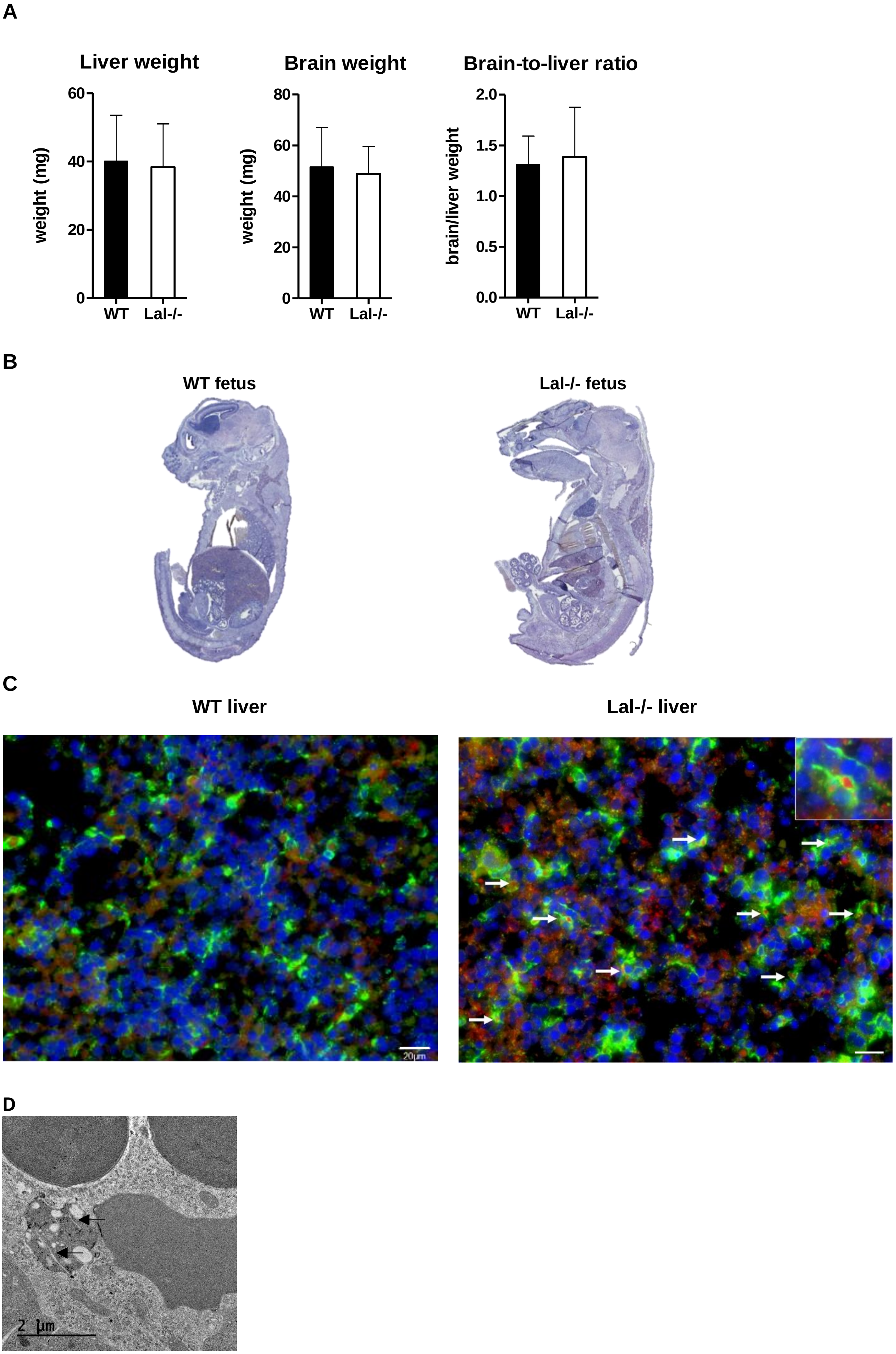
A
Liver weight
weight (mg)
60
40
20
0
WT
Lal-/-
Brain weight
weight (mg)
80
60
40
20
0
WT
Lal-/-
Brain-to-liver ratio
brain/liver weight
2.0
1.5
1.0
0.5
0.0
WT
Lal-/-
B
WT fetus
Lal-/- fetus
C
WT liver
Lal-/- liver
20µm
D
2 µm

E

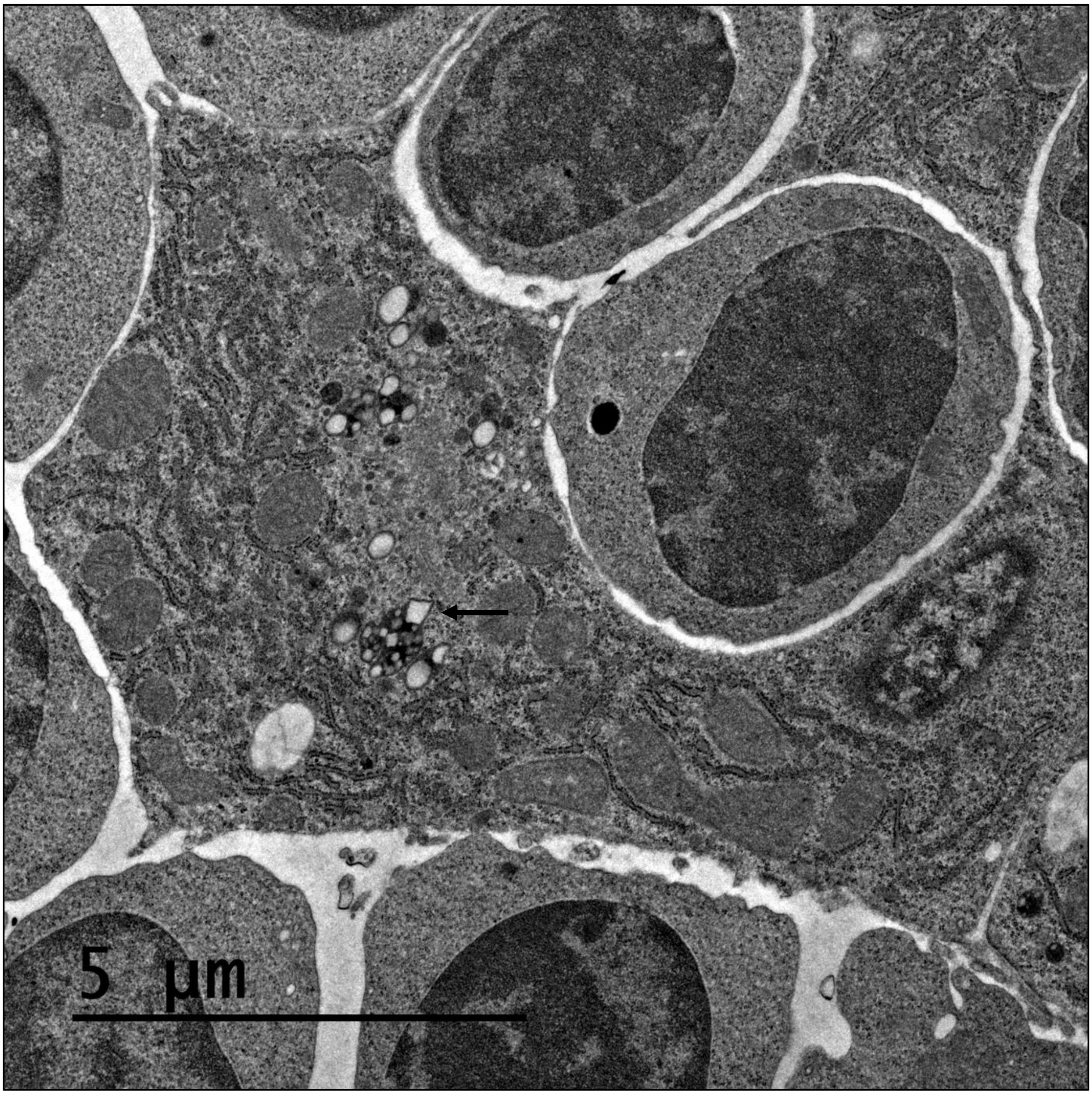

Figure S3. Increased abundance of lysosomal lipids in fetal Lal-/- livers. Fetuses were isolated from WT and Lal-/- mice on day 19 of pregnancy. (**A**) Fetal liver and brain weights and brain-to-liver ratio. Data represent mean values + SD (n = 25 WT / 29 Lal-/-). (**B**) ORO staining of a whole-body WT and Lal-/- fetus. (**C**) Immunofluorescent staining of lysosomes (LAMP1, green), neutral lipids (ORO, red), and nuclei (DAPI, blue) in fetal liver sections; white arrows indicate lipids co-localizing with lysosomes. Magnification, 40x; Scale bar, 20 µm. (**D, E**) Electron micrographs of Lal-/- fetal liver; black arrows indicate CE crystals. Scale bars, (**D**) 2 µm, (**E**) 5 µm.

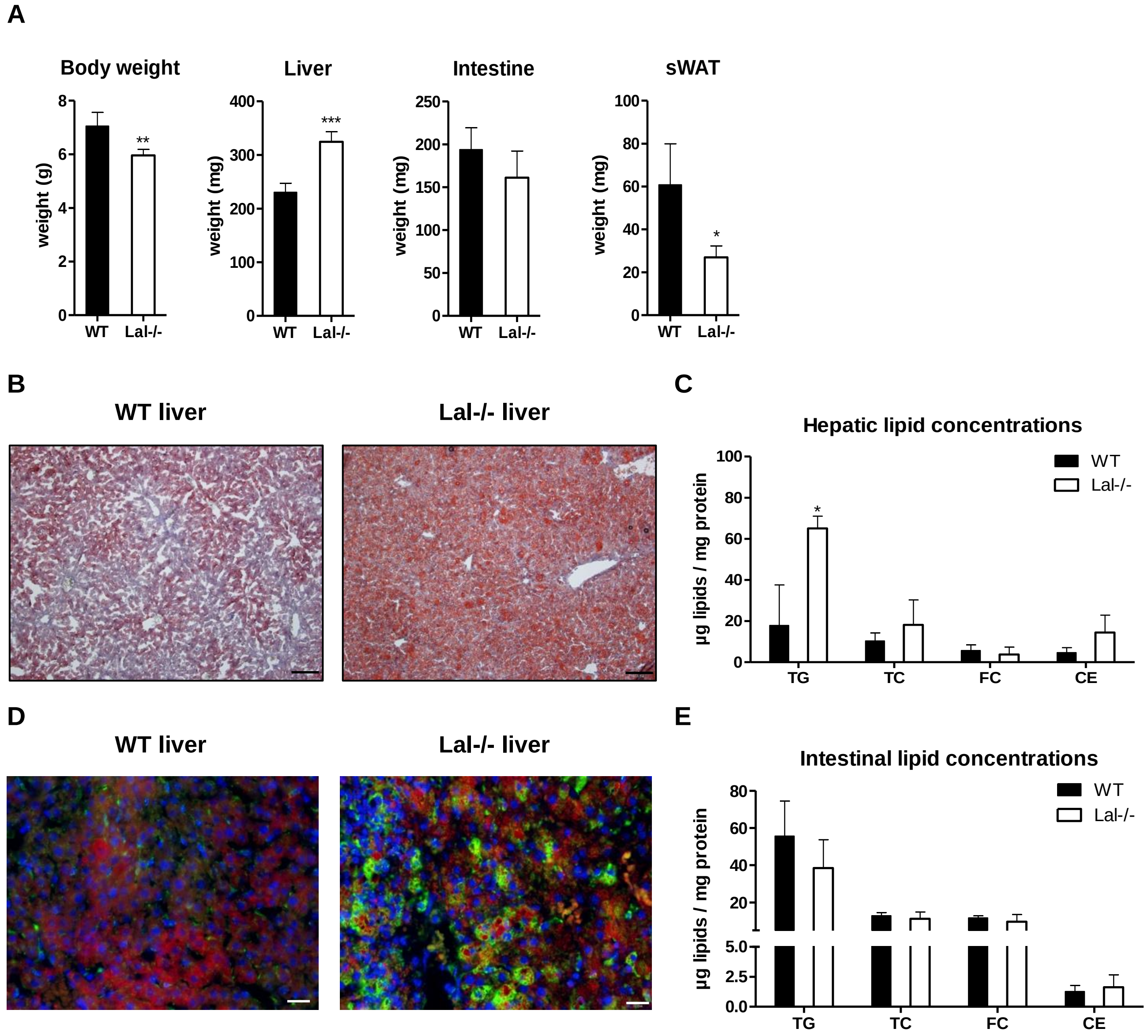

Figure S4. Lal-/- mice are severely affected by hepatic lipid accumulation already 2 weeks after birth. (**A**) Body and organ weights from 2-week-old WT and Lal-/- mice (n = 5 WT / 4 Lal-/-). (**B**) ORO staining of liver cryosections and (**C**) hepatic lipid concentrations (n = 4). (**D**) Immunofluorescent double staining for lysosomes (LAMP1, green), neutral lipids (ORO, red), and nuclei (DAPI, blue) on hepatic cryosections. Magnification, 10x; Scale bar, 100 µm. (**E**) Intestinal lipid concentrations (n = 3). Data represent mean values + SD. Statistically significant differences were calculated by two-tailed Student's t-test; * $p < 0.05$, ** $p \leq 0.\ 01$, *** $p \leq 0.001$.

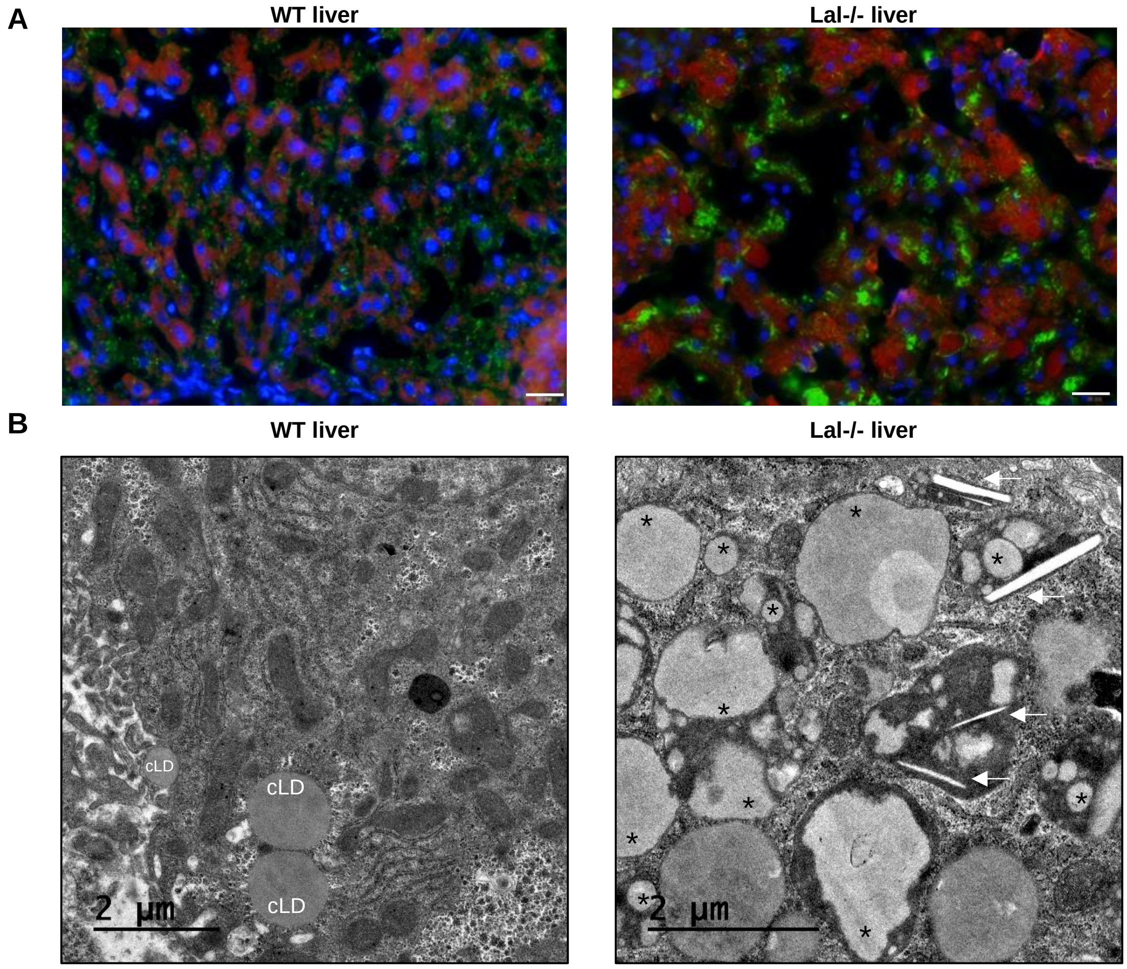

Figure S5. Lysosomal lipid accumulation in livers of 4-week-old Lal-/- mice. (**A**) Immunofluorescent staining of lysosomes (LAMP1, green), neutral lipids (ORO, red), and nuclei (DAPI, blue) on hepatic cryosections. Magnification, 20x; Scale bar, 50 µm. (**B**) Increased image size of electron micrographs of WT and Lal-/- livers; cLD, cytosolic lipid droplet; white arrows indicate lysosomal CE crystals, * indicate lysosomal lipids. Scale bar, 2 µm.
